# The Study of Titanium and Zirconium Ions in Water by MPT-LTQ Mass Spectrometry in Negative Mode

**DOI:** 10.3390/ijerph14101129

**Published:** 2017-09-26

**Authors:** Junqing Yang, Mei Zheng, Qiuju Liu, Meiling Zhu, Chushan Yang, Yan Zhang, Zhiqiang Zhu

**Affiliations:** 1 School of Chemistry and Environmental Science, Shangrao Normal University, Jiangxi 334001, China; 15779310513@163.com (M.Z.); 18270300936@163.com (M.Z.); 15720939664@163.com (C.Y.); 2 Information Engineering Faculty, Jiangxi Modern Polytechnic College, Nanchang 330095, China; yjq0930@126.com; 3 Jiangxi Key Laboratory for Mass Spectrometry and Instrumentation, East China University of Technology, Nanchang 330013, China; 18770918560@163.com (Q.L.); 15579180375@126.com (Y.Z.)

**Keywords:** microwave plasma torch, ambient mass spectrometry, titanium, zirconium

## Abstract

Microwave plasma torches (MPTs) can be used as simple and low power-consumption ambient ion sources. When MPT-mass spectrometry (MPT-MS) is applied in the detection of some metal elements, the metallic ions exhibit some novel features which are significantly different with those obtained by the traditional inductively coupled plasma (ICP)-mass spectrometry (ICP-MS) and may be helpful for metal element analysis. As the representative elements of group IVA, titanium and zirconium are both of importance and value in modern industry, and they have impacts on human health. Here, we first provide a study on the complex anions of titanium and zirconium in water by using the MPT as ion source and a linear ion trap mass spectrometer (LTQ-MS). These complex anions were produced in the plasma flame by an aqueous solution flowing through the central tube of the MPT, and were introduced into the inlet of the mass spectrometry working in negative ion mode to get the feature mass spectrometric signals. Moreover, the feature fragment patterns of these ions in multi-step collision- induced dissociation processes have been explained. Under the optimized conditions, the limit of detection (LOD) using the MS^2^ (the second tandem mass spectrometry) procedure was estimated to be at the level of 10 μg/L for titanium and 20 μg/L for zirconium with linear dynamics ranges that cover at least two orders of magnitude, i.e., between 0–500 μg/L and 20–200 μg/L, respectively. These experimental data demonstrated that the MPT-MS is a promising and useful tool in field analysis of titanium and zirconium ions in water, and can be applied in many fields, such as environmental control, hydrogeology, and water quality inspection. In addition, MPT-MS could also be used as a supplement of ICP-MS for the rapid and on-site analysis of metal ions.

## 1. Introduction

Titanium and zirconium are both important transition metal elements and have been extensively used in modern industry, scientific research, daily life and health applications [1,2,3,4]. Titanium often appears in high-powered alloys. Zirconium can be used as a strategic metal with good plasticity and strong resistance to corrosion. Meanwhile, with the wide use of a large number of materials containing zirconium or titanium, these metals inevitably enter the environment, drinking water and living organisms. Thus developing the simple and convenient detection methods for these metal elements will be of great significance for environmental protection and further studying the role of titanium and zirconium in new application fields.

The analytical methods for metal element analysis, including elements the titanium and zirconium, are mainly traditional ones, including inductively coupled plasma atomic emission spectrometry (ICP-AES) [5,6,7], spectrophotometry [8,9], atomic absorption spectrometry (AAS) [10,11], polarographic catalytic wave[12,13] and inductively coupled plasma-mass spectrometry (ICP-MS) [14,15,16]. ICP-MS is a quite sensitive elemental analysis method and can determine the contents of titanium and zirconium. Nevertheless, in the current field analytical sense, ICP-MS is limited due to the general essential tedious chemical pretreatment, expensive and cumbersome equipment [17,18,19]. Microwave plasma torch (MPT)-mass spectrometry is one of the recently developed ambient mass spectrometric techniques with numerous merits. The construction of this device is simple, as is the operation [20]. The power dissipation of MPT is usually low, less than 200 W, thus it is promising for use in the coming portable analysis fields. The plasma technique possesses strong excitation ability and good universality, hence MPT has been used widely as a powerful light source in spectrometric instruments [21,22,23,24,25,26], and even then been applied in the direct analysis of solid samples [27]. Our previous studies have showed that the MPT, as an ambient ion source, coupled with a linear ion trap mass spectrometer, can be a potential analytical tool in various fields, especially in the analysis of metal elements [17,18,19,20,28]. By adopting a desolvation unit, and injecting aqueous samples through the central-tube of the MPT unit, MPT-mass spectrometry has been applied to detect directly and sensitively many kinds of metal elements in water without tedious sample pretreatment procedures [29,30,31]. Meanwhile, the positive and negative modes of the LTQ (linear ion trap) mass spectrometer can both couple with the MPT source to perform the detection of metal elements. However, for transition metal elements, the negative mode usually exhibits lower background and simpler spectral structure [28] due to the fact that less complex metallic anions (containing several OH and H_2_O groups) produced in the MPT plasma are superposed with their corresponding isotopes, such as in the typical examples, zinc and cadmium [17]. Moreover, some transition metal elements, including titanium, zirconium and noble metals, have no obvious signal in positive mode. Although the mechanism is unknown till now, we have made efforts to study almost all transition metal elements and summarize phenomenologically the rules of their ion formation, and those results showed that MPT-mass spectrometry can be used as a supplement to ICP-MS and has potential applications in field analysis.

To further expand the applications of MPT-MS in metallic element determination in water, especially in the detection of titanium and zirconium, in-depth studies of the features of the MPT mass spectra of titanium and zirconium are necessary. This article presents the MPT mass spectra of titanium and zirconium in negative mode of a linear ion trap (LTQ) mass spectrometer, and explains the formation rule of the ions of titanium and zirconium complexes characterized by multistage collision-induced dissociation (CID) experiments. Moreover, the first results show that the LOD (limit of detection) for the detection of titanium and zirconium in water is at the level of 10^−5^ g/L. These results establish a basis for the practical application of MPT-mass spectrometry in the fields of environment control and water quality in section for the elements titanium and zirconium.

## 2. Materials and Methods

### 2.1. Materials and Reagents

The 2450 MHz microwave field was provided by a microwave generator (YY1–50 W-2450) which was purchased from the Nanjing Electronic Technology Co., Ltd. (Nanjing, China). The MPT was provided by the Yu group at Jilin University. Titanium and zirconium standard solutions (1000 μg/mL titanium and zirconium in 1.0 mol/L HNO_3_) were purchased from the General Research Institute for Nonferrous Metals (Beijing, China). Deionized water was manufactured in the chemistry facilities in Shangrao Normal University. These aqueous samples were directly analyzed by the MPT mass spectrometer without any other pretreatment except signal attenuation.

### 2.2. Experimental Conditions

The MPT device was described previously [32,33]. In brief, the MPT consists of three concentric tubes, including an outer tube for microwave input, the central tube for carrying the gas flow, and the intermediate tube for the supporting gas flow. This is a beneficial dual-flow system to optimize the plasma jet shape. The outer tube and intermediate tube were made of copper. The central tube was made of quartz. Sampling microwaves with a maximum power of 200 W were introduced into the intermediate tube through a coaxial cable and propagated in the annulus between the intermediate tube and outer tube.

**Figure 1:**
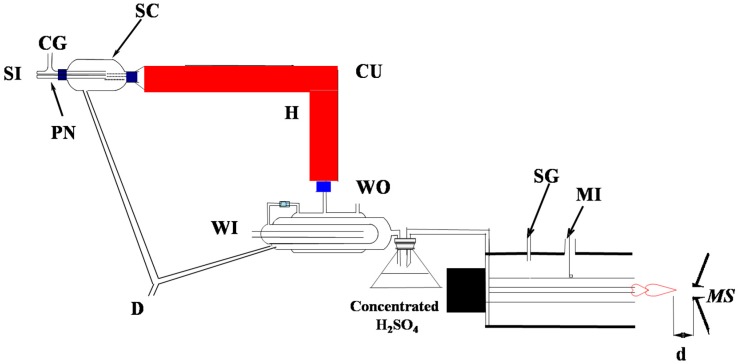
The schematic diagram of the microwave plasma torch (MPT) source coupled with the desolvation system. *Abbreviations*: MI, Microwave Input; SG, Supporting Gas Input; CG, Caring Gas; SI, Sample input; WI, Water in; WO, Water out; PN, Pneumatic Nebulizer; CU, Condenser Unit; SC, Spray Chamber; D, Drain; H, Heater; d: (the distance between the tip of MPT plasma and the inlet of LTQ); MS: (LTQ mass spectrometer).

Experiments were carried out using a LTQ-XL mass spectrometer (Finnigan, San Jose, CA, USA) equipped with the MPT ion source and the home-made pneumatic nebulization sampling system, as shown in Figure 1. The LTQ mass spectrometer was set to work in negative-ion detection mode. MS spectra were recorded in the range of 230–270 *m/z* and280–450 *m/z* for titanium and zirconium, respectively. The temperature of the ion-transport capillary was 150 °C. High purity argon gas (purity ≥ 99.999%) from a gas cylinder was divided into two routes, one of which was used for nebulizing the sample solution and carrying the dry aerosol into the central tube of MPT; and the other route was used as the supporting gas of MPT. The MPT assembly was mounted on a 3-D adjustable stage and produced stable visible and cone-shaped plasma jet on the top opening of the MPT. The distance between the tip of plasma and the MS inlet was optimized to be 1.5 mm. The full scan mass spectra were recorded using the Xcalibur software of the LTQ-MS instrument. In collision-induced dissociation (CID) experiments, the isolation width and activation time were set at 1.2 Da and 30 ms, respectively. CID was set with 30% collision energy, and other parameters were automatically optimized by the LTQ-MS system. All the mass spectra were recorded with an average duration time of 0.2 min, followed by background subtraction.

## 3. Results and Discussion

### 3.1. The MPT Mass Spectra of Titanium

**Figure 2:**
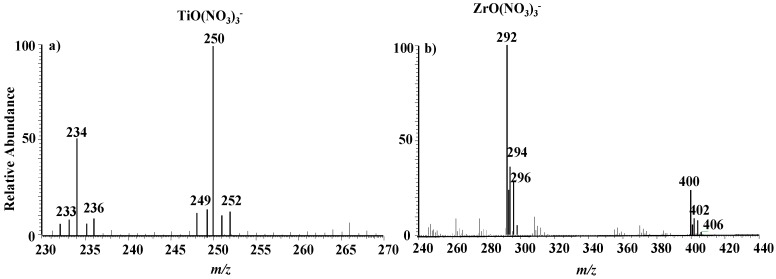
The negative-mode MPT mass spectra. (**a**) titanium; (**b**) zirconium.

In the LTQ negative working mode, the characteristic mass spectrum of titanium in the range of *m/z* 230–270 Th can be obtained with a 500 μg/L titanium standard solution, as shown in Figure 2a. Obviously, there are two similar mass spectral bands, with main peaks at *m/z* 234 and 250, respectively. Each spectral band comprises more than five peaks and the corresponding differences of the five peaks in these two spectral bands are 16 Th, i.e., the small band is the result of the large band losing an atom of oxygen. This mass spectral feature shows preliminarily that these signals are owing to the element titanium. In fact, titanium is a polyisotopic element, having five natural stable isotopes. The upper most one is ^48^Ti, with an abundance of 73.8%; the other four are ^46^Ti, ^47^Ti, ^49^Ti and ^50^Ti, accounting for 8.0%, 7.3%, 5.5% and 5.4%, respectively. Therefore, the theoretical intensity ratio of the five natural isotopes from left to right is11:10:100:8:7. However, the intensity ratio of the five peaks in the band at *m/z* 250 in turn is 13:17:100:12:18. These two ratio vales agree well. In the other aspect, based on our previous studies on MPT-mass spectrometry, the mass spectral peak of *m/z* 250 can be easy assigned to (TiO(NO_3_)_3_)^−^ and the peak at *m/z* 234 to (Ti(NO_3_)_3_)^−^.

**Figure 3:**
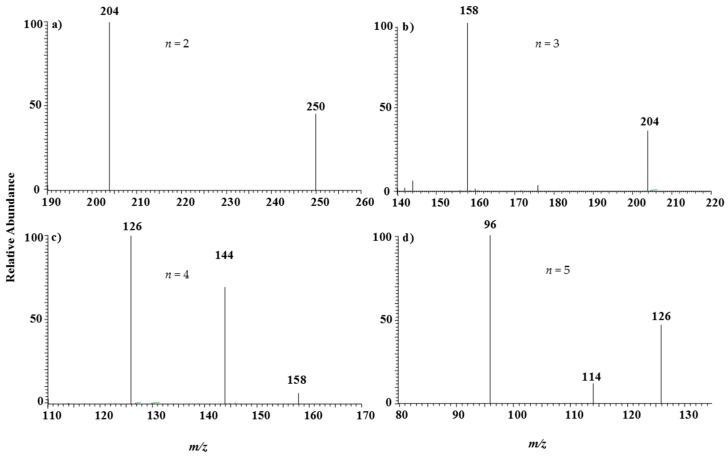
MS^n^ LTQ mass spectra (the tandem linear ion trap mass spectra) of titanium showing the dissociation sequence of the precursor ions of *m/z* 250, *n* = 2–5 in **a**–**d**, respectively.

To further confirm the signal shown in Figure 2a, tandem mass spectrometry experiments are necessary. Figure 3 showed the MS^n^ mass spectral sequence of the *m/z* 250 ions. Figure 3a is the MS^2^ mass spectrum, where the precursor ions of *m/z* 250 produce a single fragment at *m/z* 204 by the loss of NO_2_, a 46-Da group.

The NO_2_ group had been found frequently in the collision-induced dissociation processes of nitrate complexes of many transition metal elements in both positive and negative mode. The fragments of m/z 204 can be further dissociated to generate mainly the sub-fragment at m/z 158 by losing a second NO_2_ group, as shown in Figure 3b. The following dissociation of the subfragment of m/z 158 yields ions at m/z 144 and m/z 126 by expelling a 14-Da group and a 32-Da group, respectively (Figure 3c). The 14-Da group is a N atom and the 32-Da group is O_2_ which both originate from the residual NO_3_ groups by loss of the NO_2_ group. The ions of m/z 126 can still be further fragmented to produce the final ions of m/z 96. This dissociation sequence proves that the ions of m/z 250 are (^48^TiO(NO_2_)_3_)^−^ so then the precursor ions of m/z 234 are doubtlessly (^48^Ti(NO_2_)_3_)^−^. The final ions of m/z 96 cannot be further dissociated since it can be assigned to (^48^TiO_3_)^−^ from the above deduction. This structure is compact and hard to fragment.

### 3.2. The MPT Mass Spectra of Zirconium

**Figure 4:**
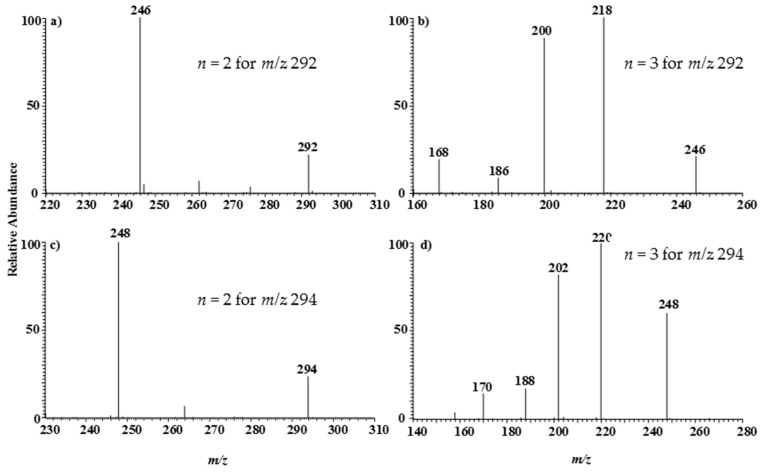
MS^n^ LTQ mass spectrometry (the tandem linear ion trap mass spectra) of zirconium showing the dissociation sequence of the precursor ions of *m/z* 292 (**a**,**b**) and 294 (**c**,**d**), respectively.

Figure 2b shows the MPT-LTQ mass spectrum of zirconium complex anion obtained by using a 1 mg/L zirconium standard solution. Similarly, there are two analogue spectral bands with central locations at *m/z* 292 and *m/z* 400. The band of *m/z* 292 includes five evident peaks located at *m/z* 292, 293, 294, 296 and 298, corresponding to the five natural isotopic distribution: ^90^Zr(51.45%), ^91^Zr(11.22%), ^92^Zr(17.15%), ^94^Zr(17.38%), ^96^Zr(2.80%).The values in the parentheses represent the relevant abundance. The intensity ratio of these five peaks in the band of *m/z* 292 is 100:24:36:28:5, very close to the theoretical value 100:22:33:34:5. Therefore, this band can be assigned preliminarily to (ZrO(NO_3_)_3_)^−^, which may be the dissociated product of the other band of *m/z* 400, corresponding to (Zr(NO_3_)_5_)^−^. This inference can be further supported by the following series tandem mass spectrometry experiments of precursor ions *m/z* 292 and 294, as shown in Figure 4a–d.

In Figure 4a, the ion *m/z* 292 dissociated a NO_2_ to generate the fragment *m/z* 246, and the fragment *m/z* 246 further produce the sub fragments *m/z* 218 and 200 by losing a 28-Da (2N atoms) and a 46-Da (NO_2_), respectively. The weak peaks of *m/z* 186 and 168 may be the double-products of *m/z* 218 and 200 by throwing the residual O_2_ groups. Figure 4c,d, showing the dissociation mechanism of the precursor ion *m/z* 294, are analogous completely to Figure 4a,b, which exhibits the fine isotopic characters.

From the MPT mass spectra of titanium and zirconium, the common features can be concluded as follows: these IVB elements generate complex anions in the form of (M(NO_3_)_n_)^−^ in the MPT plasma, here M represents the metal element. This feature is similar with that of molybdenum [16], a nearby transition metal element. However, this complex anion often dissociates to produce (M(NO_2_)(NO_3_)_n−1_)^−^ through the fragmentation of a NO_3_^−^ ligand and is a little different from the familiar transition metal elements, such as copper, zinc and so on. Obviously, the formation rules of transition metal elements in negative mode MPT-mass spectrometry are closely related to the location in the periodic table.

### 3.3. Semi-Quantitative Analysis

**Figure 5:**
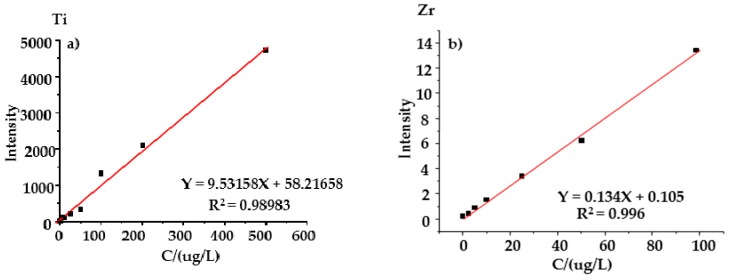
The standard curves for: (**a**) titanium and for (**b**) zirconium.

The negative mode MPT mass spectra of transition metal elements usually exhibit lower background noise and more regular mass spectral structures than positive mode ones, thus they are more suitable for qualitative and quantitative analysis [15,16,17,18,26]. To improve the sensitivity of this analysis method, the quantifications of titanium and zirconium were performed based on their respective characteristic fragments in the MPT-MS^2^ experiments of their main peaks, i.e., the fragment at *m/z* 204 for titanium and *m/z* 246 for zirconium, respectively. The plots of the measured MS^2^ fragment intensities versus the concentrations in water solutions display a good linear relationship with the linear correlation square coefficients 0.98983 for titanium and 0.996 for zirconium with the linear range of 10–500 μg/L and 20–100 μg/L, respectively, as shown in Figure 5a,b.

Based on the standard formula LOD = 3cσ/S [34], where c is the concentration of titanium or zirconium in the aqueous sample, σ is the standard deviation of all the measurements performed on the blank sample, and S is the mean value of the signals repeatedly measured, the calculated limit of detection (LOD) values for titanium and zirconium are 10 μg/L for titanium and 20 μg/L for zirconium, respectively. These quantitative data are summarized in Table 1. For easily comparison, Table 2 summarized the limit of detection values for several common analysis methods. Although the values obtained by this work were higher than that obtained with the ICP-MS and polarographiccatalytic wave methods, MPT-MS was obviously still on the same level as ICP-AES and spectrophotometry in detecting titanium and zirconium in water. Therefore, these results show that this negative mode MPT-mass spectrometry technique provides an alternative approach for field analysis and can meet the real requirements in detection of metal elements in water. In addition, the analysis of a single water sample can be finished in 5–6 min.

## 4. Conclusions

Without sample pretreatment, a direct method for detecting titanium and zirconium in water has been developed based on an MPT ion source coupled with a LTQ ion trap mass spectrometer working in the negative mode. By using pneumatic nebulization and sample injection through the central tube of the MPT device, these methods are sensitive and the detection limits for titanium and zirconium are 10 μg/L for titanium and 20 μg/L for zirconium, respectively, both at trace levels. The analysis speed is high enough to allow the direct detection of titanium and zirconium through the confidence of the characteristic peaks of the complex anions present in the MPT plasmas of titanium and zirconium within six minutes with minimum sample pretreatment. Therefore, the analysis of real samples showed that this method will have high potential applications in quality monitoring of titanium and zirconium ions in water. More promisingly, MPT-MS can be used as a supplement to ICP-MS for the detection of trace metal elements in aqueous solutions.

## Figures and Tables

**Table 1:** Summary of the performance of MPT-LTQ-MS (microwave plasma torch-linear ion trap mass spectrometry) for quantitative detection of leadin aqueous solution.

Metal Element	Linear Fitting Equation	Related R^2^	Linear Range (μg/L)	LOD (Limit of Detection) (μg/L)
Ti	*y* = 9.53158*x* + 58.2165	0.98983	10–500	10
Zr	*y* = 0.134*x* + 0.105	0.996	20–100	20

**Table 2:** Comparison of the limit of detection (LOD) for several methods in detecting the elements Ti and Zr in water.

Element	ICP-MS	ICP-AES [35]	Polarographic Catalytic Wave	Spectrophotometry	This Work
Ti	0.46	20	4	20	10
Zr	0.04	10	<10	18	20

Unit: μg/L.
